# Catheter-Deliverable Floating Hydrogels for Sustained Intravesical Drug Release

**DOI:** 10.3390/gels12080663

**Published:** 2026-07-23

**Authors:** Jing Li, Chao Ni, Sitian Li, Yutian Huang, Jun Yue

**Affiliations:** 1 School of Biomedical Engineering, Shenzhen Campus of Sun Yat-sen University, Shenzhen 518107, China; lijing385@mail2.sysu.edu.cn (J.L.); nich3@mail2.sysu.edu.cn (C.N.); list28@mail2.sysu.edu.cn (S.L.); huangyt269@mail2.sysu.edu.cn (Y.H.); 2 NMPA Center for Innovation and Research in Regulatory Science, School of Biomedical Engineering, Shenzhen Campus of Sun Yat-sen University, Shenzhen 518107, China

**Keywords:** urinary tract infection, intravesical drug delivery, floating drug delivery system, pH-responsive hydrogel

## Abstract

Recurrent urinary tract infection (rUTI) continues to pose a formidable clinical challenge, largely owing to the rapid clearance of therapeutic agents from the bladder caused by short intravesical residence time and periodic urinary voiding. Although intravesical drug delivery has emerged as a promising local therapeutic strategy, conventional liquid instillations and physically crosslinked hydrogels frequently fail to sustain structural integrity and prolonged drug release within the dynamically changing bladder microenvironment. Herein, we develop a photocrosslinkable, pH-responsive intravesical floating drug delivery system (iFDDS) for sustained antimicrobial delivery. This system is fabricated using diacrylated Pluronic F127 (F127DA) as the core network-building component. The covalently crosslinked F127DA network confers superior mechanical stability while preserving amphiphilic micellar domains that enable efficient loading of hydrophobic drugs. A tertiary amine-based pH-responsive crosslinker (CLMA) is further integrated into the hydrogel matrix, endowing iFDDS with enhanced swelling capacity under the mildly acidic microenvironment. Additionally, lyophilization-induced porous architecture reduces the apparent density of the iFDDS below that of urine, achieving stable flotation for over 48 h and effectively mitigating the risk of urinary tract obstruction. The optimized iFDDS exhibits favorable catheter deliverability, shear-thinning rheological behavior adaptable to dynamic fluid conditions, and excellent biocompatibility with bladder epithelial cells. Upon loading with rifampicin, the iFDDS demonstrates potent and sustained antibacterial efficacy against *Escherichia coli*. This study establishes a robust, environment-adaptive platform for intravesical therapy, offering a viable strategy to address the short residence time limitation of conventional formulations and improve the therapeutic management of rUTI.

## 1. Introduction

Urinary tract infection (UTI) is one of the most common bacterial infectious diseases in clinical practice and represents a substantial proportion of healthcare-associated infections, affecting a large number of patients worldwide and imposing considerable medical and socioeconomic burdens [1,2,3]. Among them, recurrent urinary tract infection (rUTI) is particularly prevalent in women. Repeated episodes of urinary frequency, urgency, dysuria, and lower abdominal discomfort not only impair daily quality of life but may also contribute to anxiety, depression, and other psychological burdens [4,5,6]. At present, antibiotics remain the mainstay for the treatment of UTI and rUTI; however, repeated or prolonged systemic administration is associated with gastrointestinal side effects, disruption of the normal microbiota, and selection of resistant bacterial strains. The increasing prevalence of antimicrobial resistance further challenges conventional systemic therapy [2,7,8]. In this context, intravesical drug delivery (IDD), which directly instills therapeutic agents into the bladder lumen through a catheter, has emerged as an important local therapeutic strategy for bladder infections and other bladder diseases because it can increase intravesical drug concentration while reducing systemic exposure [5,7,9]. Compared with oral or intravenous administration, IDD can partially bypass systemic metabolism and concentrate drugs at the bladder mucosa and lesion sites, showing therapeutic potential in bladder cancer, interstitial cystitis, and rUTI [7,9,10]. Nevertheless, the bladder is not a static drug reservoir. Continuous urine production coupled with periodic voiding results in the incessant dilution and washout of the instilled therapeutic agents, markedly limiting the effective residence time of conventional liquid formulations in the bladder [10,11,12]. In addition, tight junctions between urothelial cells, uroplakin plaques on umbrella cells, and the glycosaminoglycan (GAG) layer covering the bladder mucosa form a highly selective bladder barrier, further restricting the sustained contact and effective penetration of drugs into diseased regions [11,13,14]. Therefore, simple liquid instillation often fails to maintain sufficient local drug exposure. In clinical practice, increased instillation frequency is commonly required to compensate for insufficient efficacy, but repeated catheterization may induce urethral mucosal irritation and increase the risk of secondary infection [9,10]. Accordingly, the development of a drug delivery system capable of long-term intravesical retention, stable drug release, and reduced dosing frequency is important for improving the treatment of bladder infection-related diseases such as rUTI.

To extend the residence time of therapeutic agents in the bladder, various intravesical delivery strategies have been developed, including mucoadhesive systems, injectable hydrogels, in situ gels, nanoparticles, implantable devices, and floating drug delivery systems [9,10,13,15]. Hydrogels have attracted particular attention owing to their high water content, tunable network structures, excellent biocompatibility, and versatile drug-loading capability, making them promising platforms for localized and sustained drug delivery [16,17]. Among these, floating drug delivery systems (FDDSs) were originally developed for gastric retention. Their core principle is to maintain the overall density of a formulation below that of the surrounding liquid through low-density structures, gas generation, or porous networks, thereby allowing the formulation to remain buoyant and prolong local residence in a liquid environment [18,19]. Because a certain volume of residual urine remains after bladder voiding, the bladder provides a physiological basis for the application of FDDS. Such systems may remain suspended within the bladder lumen, reduce the possibility of material deposition at the bladder base or near the urethral outlet, and continuously release therapeutic agents [10,20]. Current intravesical floating systems mainly rely on thermosensitive gels, gas generation, or liquid–gas phase transition to provide buoyancy. For example, Lin et al. developed a Poloxamer 407/NaHCO_3_-based thermosensitive floating hydrogel that formed a gel at body temperature and generated CO_2_ through an acid-base reaction to provide buoyancy for intravesical adriamycin delivery [20,21]. Subsequently, similar NH_4_HCO_3_ gas-generating systems were used to construct CO_2_ bubble-generating floating hydrogels to reduce the risk of urinary obstruction caused by gel deposition in the bladder [22]. In addition, phase-transition materials such as perfluoropentane (PFP) have been introduced into intravesical floating hydrogels, where vaporization at body temperature produces bubbles for both hydrogel floating and visualized monitoring [23]. These studies provide an important foundation for intravesical floating drug delivery; however, current systems still have several limitations. On the one hand, some hydrogels rely mainly on thermosensitive hydrophobic association to form physical networks, and their mechanical stability and long-term structural integrity under cyclic bladder filling, voiding, and fluid disturbance remain to be improved [20,24]. On the other hand, for floating systems that rely on CO_2_ or other gas generation, buoyancy formation can be influenced by urine pH, gas generation rate, hydrogel network strength, and environmental disturbance, potentially leading to unstable floating behavior, local microenvironmental fluctuation, or insufficient gel integrity [22,23,25]. Therefore, the fabrication of an intravesical floating drug delivery system that integrates a stable network structure, durable buoyancy, controllable drug release, and good adaptability to the bladder environment remains a considerable challenge [26].

In this study, we rationally engineered a photocrosslinked hydrogel-based intravesical floating drug delivery system (iFDDS) to achieve long-term intravesical retention and sustained antibacterial drug release within the bladder. Diacrylate-terminated Pluronic F127 (F127DA) was chosen as the primary network-forming building block. As a classic PEO-PPO-PEO triblock copolymer, Pluronic F127 is capable of self-assembling into micelles in aqueous solutions via hydrophilic–hydrophobic interactions [21,27,28]. After acrylate functionalization, F127DA retains the classic PEO-PPO-PEO triblock copolymer structure of the original Pluronic F127. In aqueous solutions, its hydrophobic PPO segments aggregate to form the core, while the hydrophilic PEO segments extend outward, spontaneously assembling into stable amphiphilic micelles. This provides an excellent microenvironment for the solubilization and loading of hydrophobic drugs. On this basis, a three-dimensional porous architecture was constructed in the hydrogel rods via lyophilization to reduce the apparent density of the system and endow it with stable buoyancy, thereby enabling prolonged retention in the residual urine of the bladder. To further modulate drug release kinetics, a tertiary amine-functionalized pH-responsive crosslinker (CLMA) was incorporated into the hydrogel network. CLMA maintains a stable crosslinked structure under neutral or weakly alkaline conditions. In contrast, the protonation of tertiary amine groups under acidic conditions amplifies the internal electrostatic repulsion of the network, triggers hydrogel swelling and network expansion, and ultimately facilitates drug release. Given the tight correlation between rUTI progression and bacterial proliferation, the antibiotic rifampicin was loaded into the iFDDS to integrate intravesical floating retention with sustained antibacterial drug delivery. Following this rational design, we systematically investigated the synthesis process, structural characteristics, micromorphology, catheter deliverability, rheological performance, floating stability, pH-responsive swelling behavior, drug release profiles, biocompatibility, and antibacterial efficacy of the iFDDS. This work provides a versatile material delivery platform for the long-term treatment of bladder infection-associated disorders.

## 2. Results and Discussion

### 2.1. Preparation and Characterization of iFDDS Hydrogel Rods

Pluronic F127 diacrylate (F127DA), a triblock copolymer composed of hydrophilic poly(ethylene oxide) (PEO) and hydrophobic poly(propylene oxide) (PPO) segments, served as the primary network-forming component in this hydrogel system. The synthetic route and NMR spectra are provided in Figure S1 of the Supporting Information. Its concentration directly influenced the crosslinking density and microstructure of the final network. In this study, F127DA aqueous solutions with concentrations of 5%, 7.5%, and 10% (*w*/*v*) were prepared and crosslinked via photopolymerization in cylindrical molds under the same ultraviolet irradiation conditions (365 nm, 10 mW/cm^2^, 5 min) to obtain cylindrical hydrogel samples.

**Figure 1:**
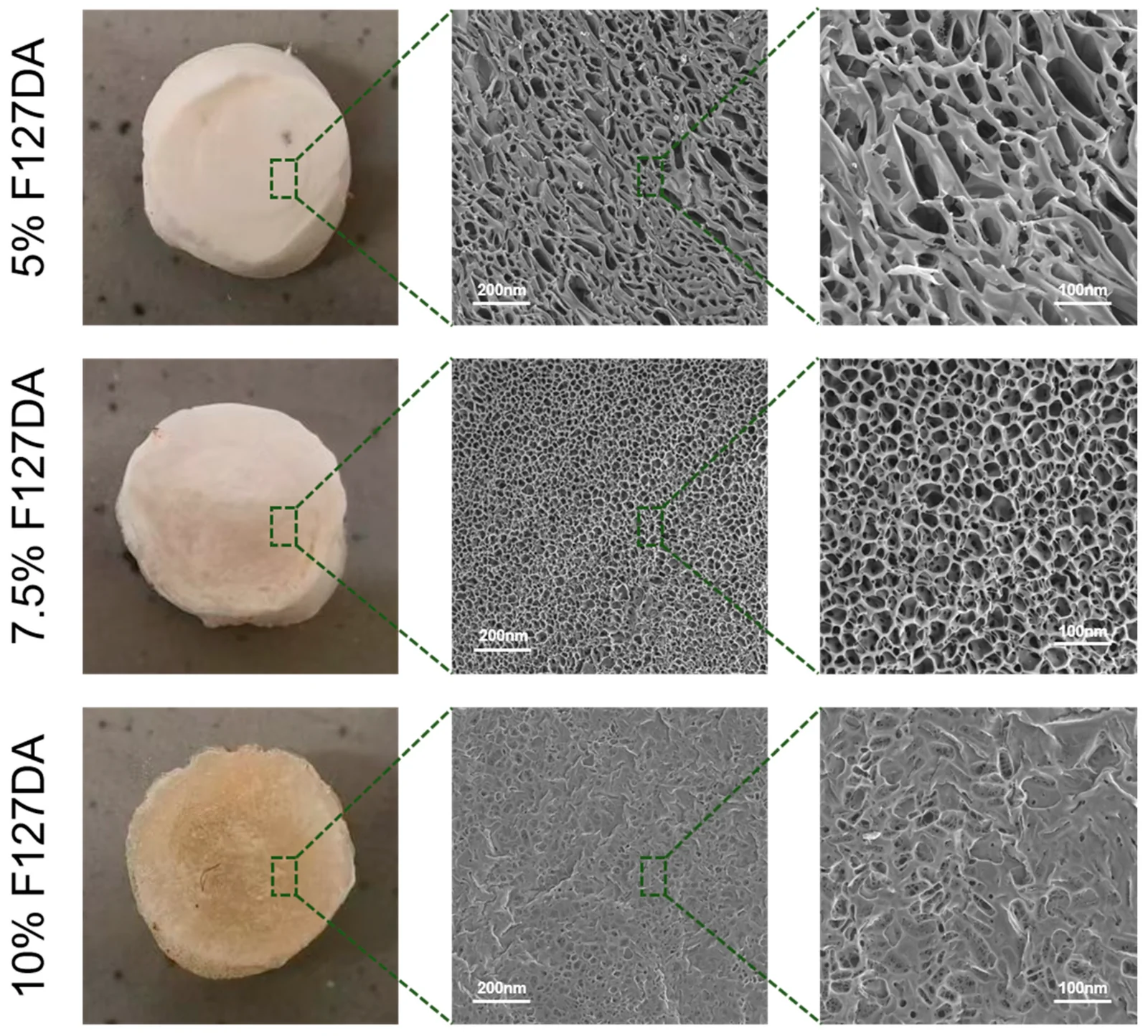
Morphological observations of F127DA hydrogels with different F127DA compositions. Representative digital photographs and SEM images of lyophilized F127DA hydrogels prepared from 5%, 7.5%, and 10% (*w*/*v*) F127DA precursor solutions.

After lyophilization, the hydrogel samples were examined by scanning electron microscopy (SEM) at different magnifications to evaluate their surface morphology. As shown in Figure 1, all groups exhibited open porous networks with continuous microchannels. As the F127DA concentration increased from 5% to 10%, the average pore size markedly decreased, which was consistent with the increase in crosslinking sites and the formation of a denser network at higher polymer concentrations. Compared with the 5% and 10% F127DA hydrogels, the 7.5% F127DA group showed a more appropriate pore size and the most uniform pore distribution. The pore size and pore number of the hydrogels are closely associated with the overall density of the system. Therefore, selecting an appropriate F127DA concentration is beneficial for reducing the density of the hydrogel rods and enhancing their buoyancy in aqueous environments. This structure-property relationship provided an experimental basis for the subsequent design of the floating delivery system.

### 2.2. Catheter Deliverability and Floating Performance of iFDDS Hydrogel Rods

**Figure 2:**
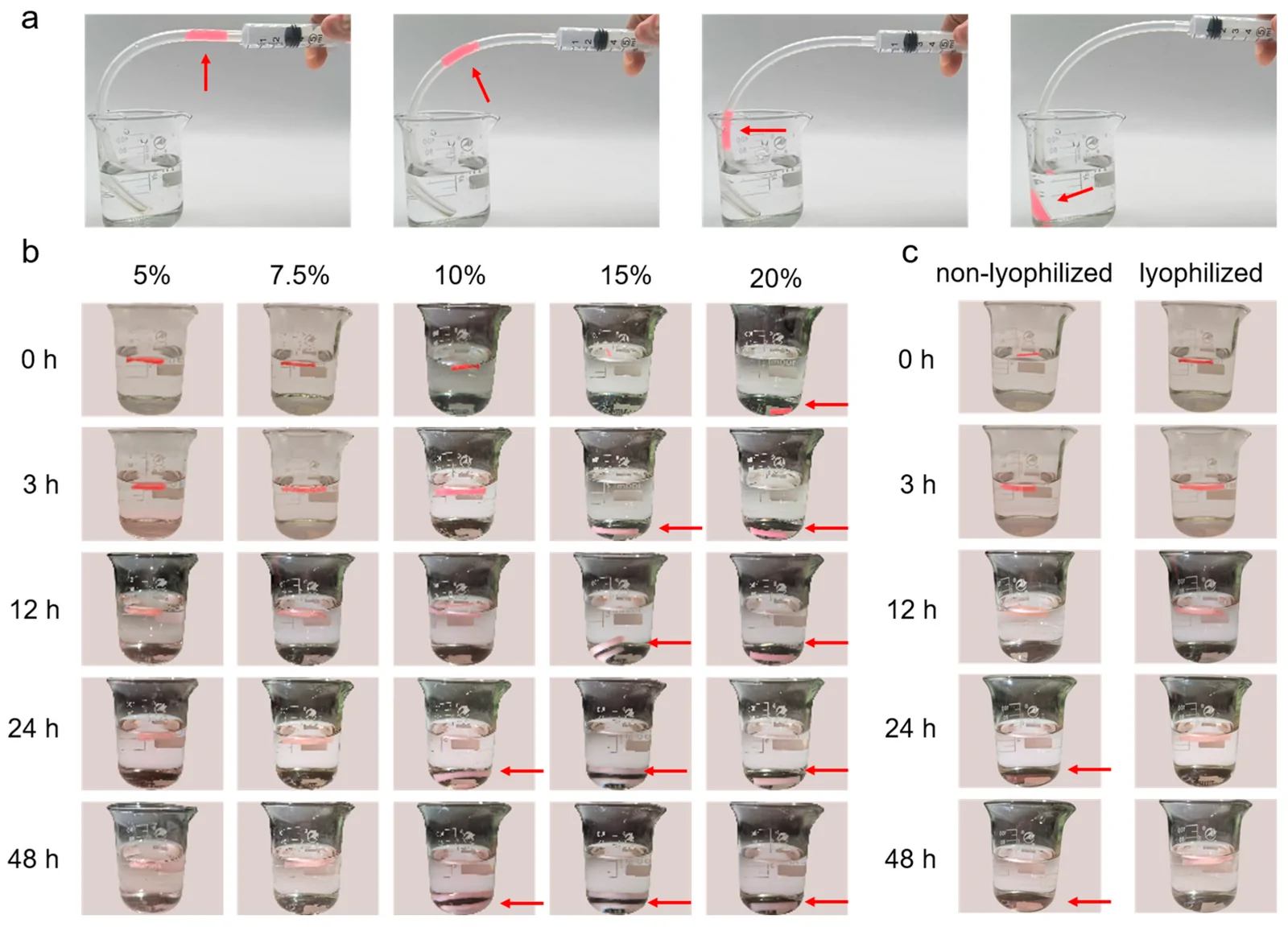
Catheter deliverability and buoyancy performance of iFDDS hydrogel rods. (**a**) Representative photographs showing the catheter-assisted delivery process of the iFDDS hydrogel rod through a silicone tube, with red arrows indicating the position of the rod during syringe propulsion; (**b**) Time-lapse floating behavior of lyophilized F127DA hydrogel rods with varying polymer concentrations (5%, 7.5%, 10%, 15%, and 20% *w*/*v*) in PBS at 37 °C over 48 h; (**c**) Comparison of the floating behavior between non-lyophilized and lyophilized 7.5% F127DA hydrogel rods. Red arrows indicate the sedimentation of samples.

Because the iFDDS is intended to be delivered into the bladder through a catheter in clinical use, evaluating its catheter deliverability is critical for assessing system feasibility. A silicone tube with an inner diameter of 4 mm was used as a model catheter. Hydrogel rods prepared with 7.5% F127DA were lyophilized and then immersed in phosphate-buffered saline (PBS, pH 7.4) for 10 min to fully hydrate the surface, thereby reducing frictional resistance and improving lubrication. The delivery setup consisted of a silicone tube, a syringe, and a beaker containing liquid. One end of the silicone tube was connected to the syringe, whereas the other end was immersed in the liquid in the beaker. During the experiment, a stable and uniform force was applied to the syringe to continuously transport the hydrogel rod through the silicone tube. As shown in Figure 2a, the red arrows indicate the position of the hydrogel rod. Under a relatively low pneumatic driving force, the hydrogel rod moved smoothly through the tube and maintained its structural integrity without obvious jamming, obstruction, or fracture. These results demonstrate that the F127DA hydrogel system possesses favorable catheter deliverability and can meet the operational requirements of commonly used clinical catheters. This experiment verifies the practical applicability of the floating delivery system in an intravesical delivery scenario and provides technical support for subsequent translational and preclinical evaluation.

Floating performance is one of the key indicators for determining whether the drug delivery system can achieve long-term residence in the bladder. We systematically investigated the effects of F127DA concentration and lyophilization on hydrogel buoyancy. As shown in Figure 2b, red arrows indicate hydrogel samples that had sedimented. The floating duration decreased as the F127DA concentration increased. When the concentration was increased to 10% and 15%, the samples sedimented within 24 h and 3 h, respectively, whereas the 20% group lost floating ability at the initial stage of the experiment. In contrast, the low-concentration groups (5% and 7.5%) exhibited excellent floating stability and remained buoyant for more than 48 h. This concentration-dependent behavior provided experimental guidance for formulation optimization. Finally, through quantitative profiling of apparent density, real-time buoyancy, and water uptake (Figure S2), we have confirmed that the hydrogel rods float by virtue of a bulk density that remains intrinsically below that of the immersion medium, with no progressive degradation of buoyancy over time.

The floating performance of lyophilized and non-lyophilized samples was further compared (Figure 2c). Because no external porogen was introduced into the system, the porous structure mainly originated from the F127DA crosslinked network and the voids left by ice crystal sublimation during lyophilization. The non-lyophilized hydrogels sedimented within 24 h, whereas the lyophilized samples maintained stable floating for more than 48 h. These results indicate that lyophilization under relatively low crosslinking density conditions favors the formation of a more developed porous structure, decreases the overall density of the system, and significantly improves floating performance.

### 2.3. Swelling Behavior of iFDDS Hydrogel Rods

The small-molecule crosslinker CLMA synthesized in this study contains both tertiary amine groups and reactive double bonds. The synthetic route and NMR spectra are provided in Figure S3 of the Supporting Information. The tertiary amine groups provide pH-responsive swelling capability, whereas the double bonds participate in photopolymerization and introduce additional covalent crosslinking sites into the hydrogel network. Because these two effects can counterbalance each other, it was necessary to systematically investigate the influence of CLMA concentration on the swelling behavior of the hydrogel rods.

**Figure 3:**
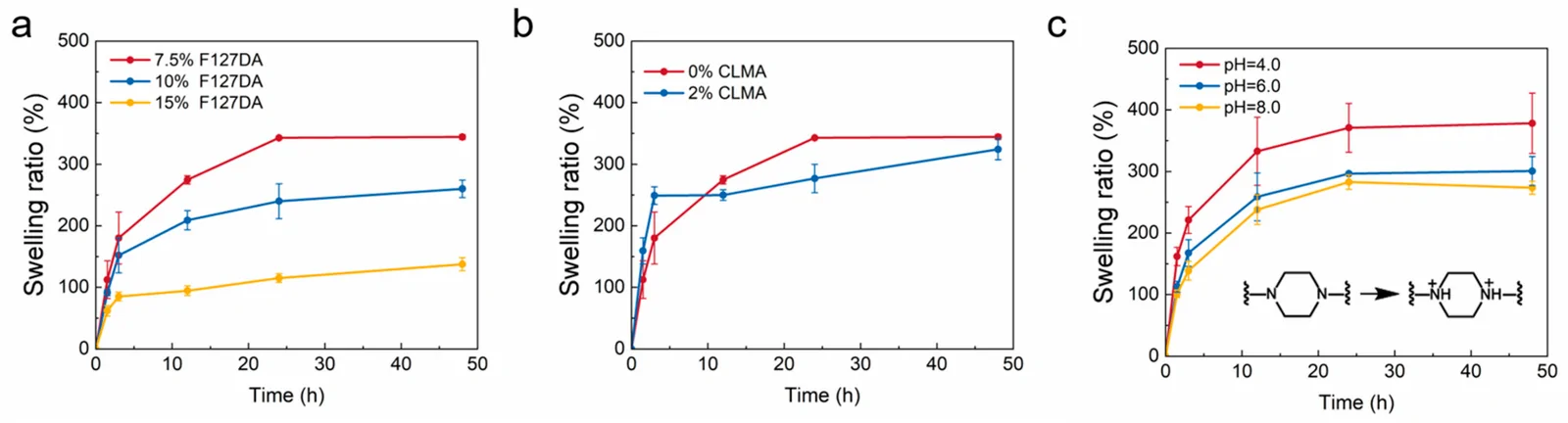
Swelling behavior of iFDDS hydrogel rods. (**a**) Swelling profiles of F127DA hydrogel rods prepared with different F127DA concentrations (7.5%, 10%, and 15% *w*/*v*); (**b**) Effect of incorporating CLMA on the swelling behavior of hydrogel rods in PBS (pH 7.4); (**c**) pH-responsive swelling behavior of hydrogel rods composed of 7.5% F127DA and 1% CLMA in PBS at pH 4.0, 6.0, and 8.0. The inset illustrates the protonation of tertiary amine groups in CLMA under acidic conditions, which drives network expansion and macroscopic swelling. Data are presented as mean ± SD (*n* = 3).

First, to clarify the role of crosslinking density in swelling behavior, hydrogel samples with F127DA concentrations of 7.5%, 10%, and 15% (*w*/*v*) were prepared and immersed in PBS at the same pH. At predetermined time points (1.5, 3, 12, 24, and 48 h), the samples were removed, surface water was gently removed, and the diameter and length were measured to calculate the swelling ratio (Figure 3a). The swelling ratio of all groups increased rapidly at the early stage, followed by a slower increase and an equilibrium state at approximately 24 h. The swelling rate and equilibrium swelling ratio of the 15% group were significantly lower than those of the 7.5% group, indicating a negative correlation between crosslinking density and swelling ratio in this system.

The swelling kinetics of hydrogels with and without CLMA were then compared (Figure 3b). The two groups showed similar overall swelling trends, but the CLMA-containing samples exhibited a slightly higher swelling ratio during the early stage (before approximately 10 h). This may be attributed to the fact that protonation of tertiary amine groups had not yet reached equilibrium, and the positive charges introduced during protonation induced local network relaxation, thereby promoting initial swelling. As the swelling process continued and protonation approached saturation, the covalent crosslinking effect of CLMA became dominant and restricted further network expansion, resulting in a reduced swelling rate. Consequently, the hydrogels without CLMA showed a higher final swelling ratio.

Clinically, normal human urine generally exhibits a physiological pH of 4.6–8.0; meanwhile, urine often becomes mildly acidic (pH ≈ 5.0–6.0) under inflammatory microenvironments or due to metabolic byproducts of certain pathogens linked to recurrent urinary tract infections (rUTIs). Figure 3c schematically illustrates the pH-responsive swelling mechanism of CLMA. In acidic environments, tertiary amine groups bind H+ and become protonated, causing the network to carry positive charges. The resulting electrostatic repulsion promotes local relaxation of the crosslinked structure and macroscopically enhances hydrogel swelling. Because urine in the bladder is generally weakly acidic, this mechanism provides a suitable environmental basis for the responsive swelling of the material in vivo. To further verify the effect of pH on swelling behavior, hydrogel samples containing 1% CLMA were immersed in buffer solutions at pH 4.0, 6.0, and 8.0, and their swelling kinetics were measured (Figure 3c). All groups exhibited rapid swelling at the early stage, followed by gradual slowing and eventual equilibrium. Vertical comparison showed that both the swelling rate and equilibrium swelling ratio in the pH 4.0 group were significantly higher than those in the pH 6.0 and pH 8.0 groups. The swelling ratio exceeded 200% at 3 h and reached approximately 400% at 24 h. These results confirm that, under the same crosslinker content, stronger acidity (lower pH) leads to a higher swelling rate and equilibrium swelling ratio, validating the pH-responsive characteristics of CLMA.

### 2.4. Rheological Properties of iFDDS Hydrogels

**Figure 4:**
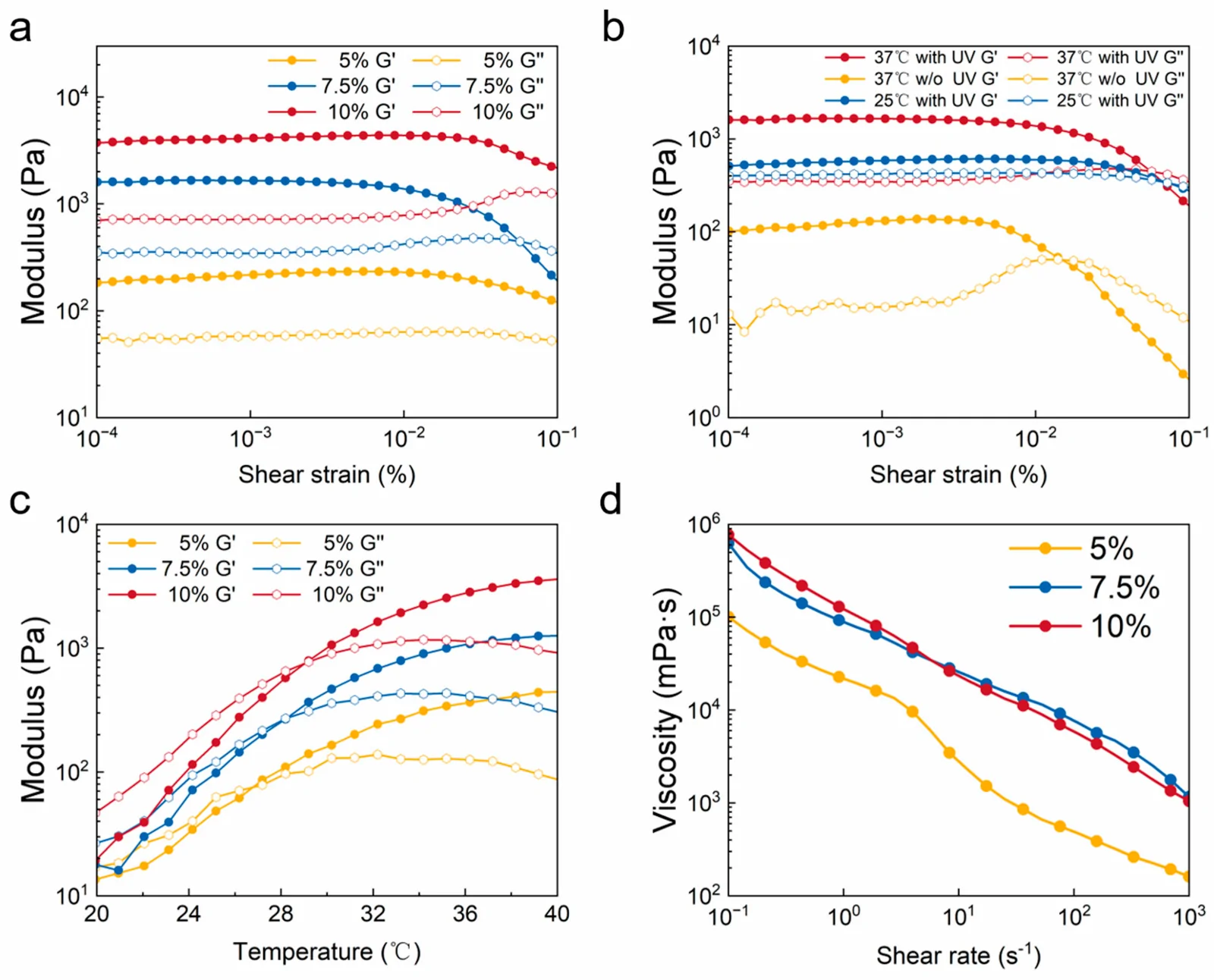
Rheological characterizations of F127DA hydrogels. (**a**) Strain-amplitude sweeps of F127DA hydrogels with different polymer concentrations (5%, 7.5%, and 10% *w*/*v*), showing the storage modulus (G′) and loss modulus (G″) as a function of shear strain; (**b**) Comparison of the viscoelastic properties of F127DA hydrogels prepared with and without UV-induced photocrosslinking at 25 and 37 °C; (**c**) Temperature-dependent rheological behavior of F127DA hydrogels with different polymer concentrations during heating from 20 to 40 °C; (**d**) Shear-thinning behavior of F127DA hydrogels with different polymer concentrations, showing viscosity changes as a function of shear rate.

To further clarify the effect of F127DA concentration on hydrogel mechanical behavior, rheological characterization was performed on F127DA hydrogels with different concentrations (5%, 7.5%, and 10%, *w*/*v*). Under a constant angular frequency, when the shear strain was gradually increased from 0.0001% to 0.1%, both the storage modulus (G′) and loss modulus (G″) of the three groups remained relatively stable, and G′ was consistently higher than G″ (Figure 4a), indicating that this strain range corresponded to the linear viscoelastic region of the hydrogels. In addition, as the F127DA concentration increased, the crosslinking density of the hydrogel increased, and the corresponding moduli also increased. The storage modulus of the 7.5% group was approximately one order of magnitude higher than that of the 5% group, suggesting that increased crosslinking density effectively enhanced the mechanical properties of the hydrogel. Traditional physically crosslinked Pluronic hydrogels rely solely on the physical packing of thermosensitive micelles to maintain their structure, making them highly susceptible to depolymerization and erosion under physiological dilution or dynamic shear. By introducing acrylate groups at the ends of the PEO chains, we endowed F127DA with UV-initiated in situ crosslinking capability. As clearly shown by the comparative rheological data in Figure 4b, the storage modulus (G′) of the hydrogel after photo-crosslinking achieved an increase of several orders of magnitude compared to that of the physically crosslinked hydrogel. Under photocrosslinking conditions, the hydrogel prepared at 37 °C exhibited a slightly higher modulus than that prepared at 25 °C. These results demonstrate that photocrosslinking markedly improves the mechanical strength of F127DA hydrogels while preserving the intrinsic thermoresponsive characteristics of Pluronic F127. This significant mechanical reinforcement enables the hydrogel to maintain its structural integrity under external fluid shear and within the complex physiological environment of bladder urine, preventing it from being rapidly washed away and thus ensuring stable localized drug delivery.

To investigate the rheological manifestation of this thermoresponsive property, temperature sweep measurements were performed within the linear viscoelastic region at a fixed shear strain of 0.01% while the temperature was increased from 20 to 40 °C (Figure 4c). As the temperature increased, the moduli of all three hydrogels increased by orders of magnitude. Before reaching the transition threshold, G″ was higher than G′, and the material mainly behaved as a viscous fluid. After exceeding the threshold, G′ became higher than G″, indicating a transition to an elastic solid-like state. In addition, the temperature threshold increased with increasing F127DA concentration, reflecting the regulatory effect of crosslinking density on the thermoresponsive transition.

Shear-thinning tests (Figure 4d) showed that the viscosity of all three hydrogels gradually decreased as the shear rate increased, and the decreasing trends were similar. At the same shear rate, the viscosities of the 7.5% and 10% groups were significantly higher than that of the 5% group, indicating that increased crosslinking density enhanced hydrogel viscosity. However, when the crosslinking density further increased to a certain level, the increase in viscosity became less pronounced. This shear-thinning behavior may improve the adaptability of the material in the bladder environment. During urination, a high shear rate can reduce hydrogel viscosity, thereby decreasing resistance to urine flow and lowering the risk of adhesion near the urethral outlet and subsequent obstruction [29].

### 2.5. Mechanical Performance and Drug Release of the iFDDS Under Simulated Bladder Conditions

**Figure 5:**
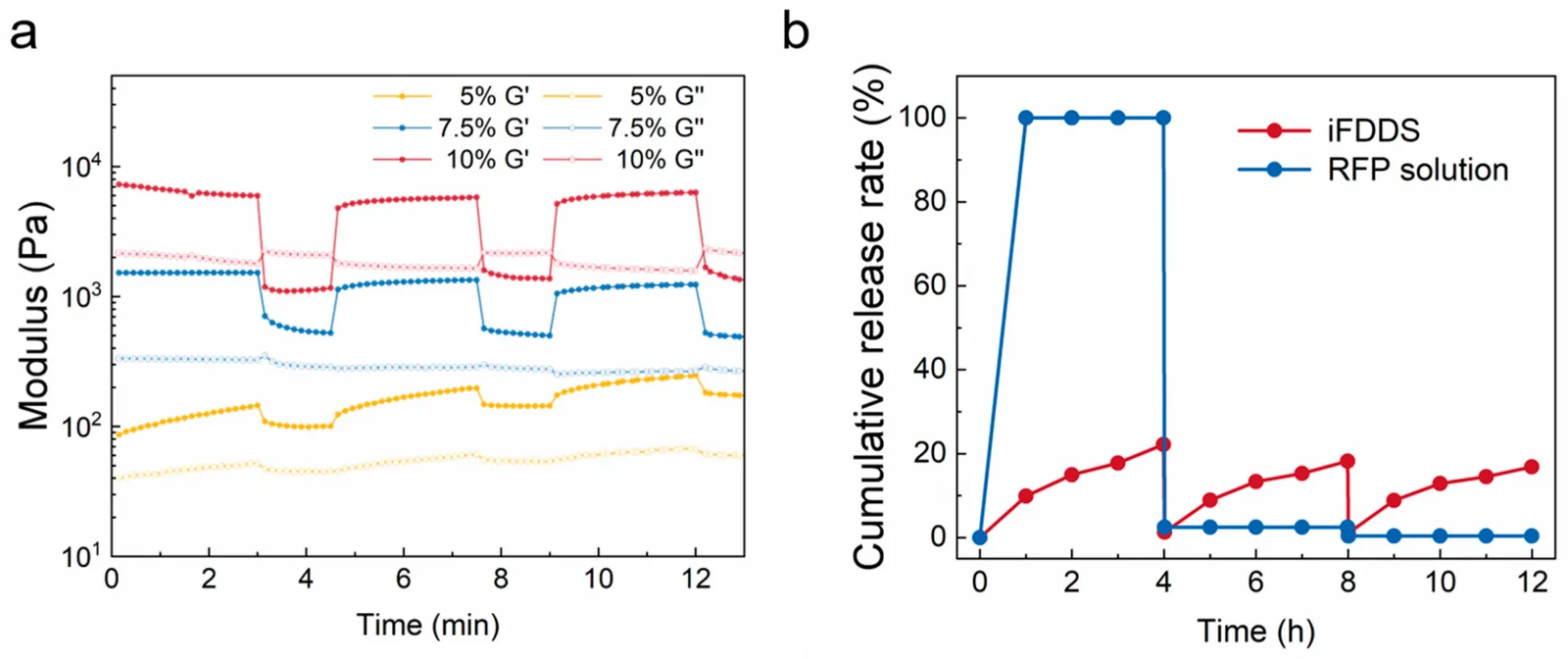
Mechanical adaptability and drug release behavior of iFDDS under a simulated bladder environment. (**a**) Time-dependent rheological response of F127DA hydrogels with different polymer concentrations (5%, 7.5%, and 10% *w*/*v*) under cyclic low- and high-strain conditions, mimicking the alternating urine storage and voiding processes in the bladder; (**b**) Cumulative release profiles of rifampicin (RFP) from iFDDS and a free RFP solution in an in vitro bladder model with repeated filling–voiding cycles. The abrupt drops in the free RFP solution group indicate rapid drug washout after simulated voiding, whereas iFDDS exhibited persistent release throughout the repeated cycles.

Because the bladder is an organ that undergoes periodic contraction and relaxation, dynamic strain tests were performed to determine whether the prepared floating drug delivery system could adapt to the mechanical environment of the bladder. Alternating shear strain was applied to simulate the changes in shear stress during urine storage and voiding (Figure 5a). During each constant-strain stage, the storage modulus (G′) and loss modulus (G″) of the three hydrogel groups remained relatively stable. When the shear strain abruptly changed from a low level to a high level, G′ decreased instantaneously, whereas G″ remained nearly unchanged or slightly increased. During the transition from low to high strain, G′ remained higher than G″ in the 5% and 7.5% groups, indicating that the hydrogels retained predominantly elastic solid-like behavior. In the 10% group, however, G′ became lower than G″ under high strain, and the material displayed a more pronounced viscous response, which may be associated with increased internal friction caused by the higher crosslinking density. After three cycles, the moduli of all groups recovered close to their initial values when the strain returned to the initial level, indicating good mechanical reversibility. Considering the magnitude and variation trend of the moduli, the 7.5% F127DA group maintained a relatively stable elastic response throughout the cyclic test without exhibiting excessive viscosity. This suggests that this formulation has advantages in maintaining structural integrity and adapting to dynamic fluid changes in the bladder, with the potential to reduce the risk of urethral obstruction caused by excessive viscosity.

To evaluate the drug release profile of the iFDDS, in vitro release was assessed under different pH conditions. We deliberately chose rifampicin as the model cargo to demonstrate proof-of-concept for accommodating hydrophobic actives, confirming the system’s ability to stably load and slowly liberate poorly soluble drugs via its crosslinked network. According to the calculation of the drug loading parameters of the drug delivery system (iFDDS) in the Supplementary Material, the encapsulation efficiency and drug loading content (DLC) of the optimized iFDDS formulation were calculated to be 72.89 wt% and 17.62 wt%, respectively. The absolute mass of rifampicin loaded in each tested single hydrogel rod is approximately 2000 g (2 mg). Detailed experimental procedures and results are provided in Figure S4 of the Supporting Information. Additionally, we have analyzed the release curve at pH 6.0 in Figure S3b using the four mathematical release models; the results demonstrate that (Figure S5), although the first-order model exhibits superior numerical fitting, the Korsmeyer–Peppas model and the derived value (0.47) collectively confirm that rifampicin release from the iFDDS is governed by the synergistic interplay of diffusion and minor network relaxation. This predominantly diffusion-driven non-Fickian regime precisely validates the rational design of the UV crosslinked network, which reconciles structural robustness with pH-responsive drug liberation. Then, to evaluate the drug release behavior of the floating drug delivery system under a complex bladder-like physiological environment, an in vitro bladder model was established. Urine medium was continuously introduced into the system at a constant flow rate using a peristaltic pump, and complete emptying was performed every 4 h for three cycles to simulate the periodic filling and voiding of the bladder. During the experiment, samples were collected every hour, and absorbance was measured to generate release profiles (Figure 5b). Compared with the direct instillation of a free drug solution, the floating drug delivery system exhibited markedly different release kinetics. In the single-dose model, the drug concentration in the medium decreased to nearly zero after the first emptying step. In contrast, the floating drug delivery system continuously released drug throughout the cyclic process and was not eliminated by urine emptying. Quantitatively, approximately 20% of the total drug loading was released during each cycle, indicating that the system could maintain a relatively stable release rate during repeated filling-emptying processes. These in vitro model results demonstrate that the floating drug delivery system can effectively prolong drug residence in the bladder and maintain sustained release under dynamic voiding conditions, showing potential advantages over single-dose administration. While the in vitro release and floating behaviors are mechanistically encouraging, translation to the clinic necessitates bridging the gap between these simplified models and the dynamic pathophysiology of the living bladder.

### 2.6. Biocompatibility and Antibacterial Performance of the iFDDS

**Figure 6:**
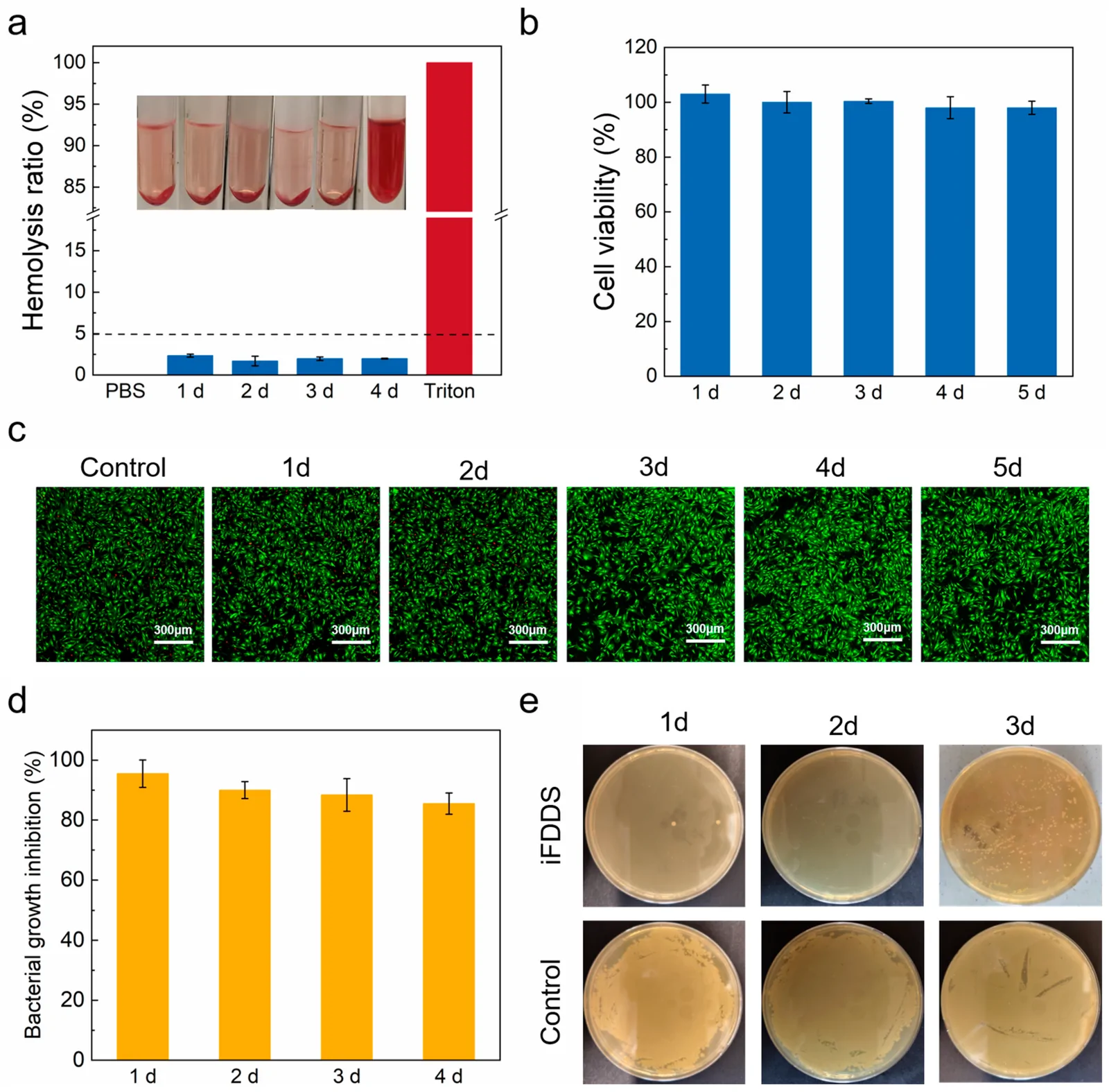
Biocompatibility and in vitro antibacterial performance of iFDDS. (**a**) Hemolysis ratios of hydrogel extracts collected at different time points, with PBS and Triton X-100 serving as the negative and positive controls, respectively. The inset shows representative photographs of red blood cells after incubation with the corresponding samples; (**b**) Viability of mouse bladder epithelial cells after incubation with hydrogel extracts collected from day 1 to day 5; (**c**) Live/dead staining images of mouse bladder epithelial cells after treatment with hydrogel extracts collected at different time points. Scale bars: 300 μm; (**d**) Bacterial growth inhibition of rifampicin-loaded iFDDS against *E. coli* after 4 days; (**e**) Representative TSB agar plate images demonstrating the antibacterial efficacy of rifampicin-loaded iFDDS after incubation with *E. coli* for 1, 2, and 3 days. Data are presented as mean ± SD (*n* = 3).

Subsequently, hemolysis and cytotoxicity assays were performed to systematically assess the biosafety of the floating drug delivery system. The hemolysis results (Figure 6a) showed that the hemolysis rates of extract solutions collected after 1, 2, 3, and 4 days were all below 3%, markedly lower than the 5% safety threshold specified in relevant standards. The hemolysis rates were close to that of the negative control (PBS) and substantially different from that of the positive control (Triton X-100). These results indicate that the floating drug delivery system has good hemocompatibility and satisfies the blood safety requirements for biomedical applications. For cytocompatibility evaluation, mouse bladder epithelial cells were used as the cell model, and the effect of hydrogel extract solutions on cell viability was quantified by the CCK-8 assay. All hydrogel preparation and crosslinking procedures were performed in a biosafety cabinet under strict aseptic conditions. As shown in Figure 6b, cell viability in all experimental groups remained around 100% relative to the control group, with no obvious decrease. Live/dead fluorescence staining was further conducted to verify this result (Figure 6c). No apparent differences were observed between the experimental groups and the control group in terms of green fluorescence intensity and distribution area, indicating that extract solutions collected at different time points did not adversely affect the survival of mouse bladder epithelial cells. These mutually supportive results demonstrate the good cytocompatibility of the floating drug delivery system and provide a safety basis for its potential clinical application. Nevertheless, hemolysis and epithelial cell viability assays only provide preliminary biocompatibility evidence and cannot fully evaluate intravesical biosafety, which is a limitation of this work. Comprehensive supplementary tests covering urothelial irritation, inflammatory response, residual photoinitiators, unreacted monomers, degradation byproducts and sterilization efficiency will be implemented in our subsequent in vivo investigations.

The in vitro antibacterial activity of the drug-loaded hydrogel was further evaluated against *Escherichia coli*, one of the most common pathogens associated with bladder infection. Diluted bacterial suspensions were co-incubated with drug-loaded hydrogels, and bacterial growth inhibition was evaluated by measuring OD_600_ values. As shown in Figure 6d, the antibacterial rate of the drug-loaded hydrogel remained above 80% at all time points. Although the inhibition rate slightly decreased with incubation time, it remained at a high level. Plate spreading experiments (Figure 6e) showed almost no bacterial growth within 48 h of incubation. After three days, the number of colonies slightly increased but remained significantly lower than that of the positive control. Collectively, the quantitative and qualitative results demonstrate that the drug-loaded hydrogel has pronounced inhibitory effects against *E. coli*, suggesting its potential for preventing or assisting the treatment of bladder infections.

## 3. Conclusions

An intravesical floating drug delivery system (iFDDS) with long-term buoyancy and pH-responsive swelling behavior was successfully constructed. iFDDS possessed a loose porous surface structure, which reduced the overall density and enabled long-term floating. The photocrosslinked network endowed the hydrogel with favorable mechanical properties, allowing it to remain stable under dynamic fluid conditions associated with periodic bladder contraction and relaxation. The system could be smoothly delivered through commonly used clinical silicone catheters and exhibited a higher swelling ratio in acidic bladder-like environments, suggesting its potential to reduce the risk of urinary obstruction. In vitro cell experiments demonstrated good biocompatibility with mouse bladder epithelial cells. After rifampicin loading, the system showed significant inhibitory effects against *E. coli*, a common pathogen in urinary tract infections. However, owing to the highly dense photo-crosslinked network, the hydrogel exhibits an extremely slow degradation rate in vitro, which represents a limitation for its clinical translation. We will address this issue by introducing degradable crosslinkers/linkages into the system in our subsequent studies. Overall, these properties suggest that the floating drug delivery system has potential clinical application value for the treatment of bladder-related diseases.

## 4. Materials and Methods

Mouse bladder transitional epithelial cells (CP-M058) were purchased from Gengzhenshi Qiyun Biotechnology Co., Ltd. (Guangzhou, China). *Escherichia coli* was purchased from Guangzhou Janda Gene Technology Co., Ltd. (Guangzhou, China). All organic solvents used in the experiments were used as received without further purification.

### 4.1. Fabrication and Morphological Characterization of Floating Hydrogels

Different amounts of F127DA were dissolved in PBS (pH 8.0) to obtain solutions with mass fractions of 5%, 7.5%, 10%, 15%, and 20% (*w*/*v*). The solutions were stored at 4 °C for 24 h to ensure complete dissolution and facilitate micelle formation. Subsequently, the pH-responsive small-molecule crosslinker CLMA (molar ratio optimized according to the desired crosslinking density) and the photoinitiator Irgacure 2959 (0.1%, *w*/*v*) were added, followed by vortexing until homogeneous, yielding hydrogel precursor solutions. The precursors were injected into silicone tube molds (inner diameter: 4 mm) and exposed to 365 nm UV light (~10 mW/cm^2^) for 5 min to complete photocrosslinking. The resulting hydrogel rods were lyophilized at −80 °C and 0.1 mbar for 48 h to obtain porous scaffolds.

Lyophilized 5%, 7.5%, and 10% samples were mounted on conductive adhesive, sputter-coated with gold, and observed using field-emission scanning electron microscopy (FE-SEM) at magnifications of ×100, ×200, ×500, and ×1000 to analyze surface morphology and pore distribution.

### 4.2. Catheter Deliverability Test

To visually evaluate catheter deliverability, rhodamine B was added to the precursor solutions. Lyophilized samples (5%, 7.5%, and 10% *w*/*v*) were immersed in PBS (pH 7.4) for 10 min to hydrate and lubricate the surface. A medical-grade silicone catheter/tubing (40 cm × 0.6 cm OD × 0.4 cm ID) was employed as both the molding template and the simulated injection conduit. A 5 mL syringe was used to propel the hydrogel rods at a constant rate. The structural fidelity of the rods during deployment was observed, and clinical operability was assessed.

### 4.3. Floating Performance Test

Floating time was the key performance parameter of the system. Two sets of experiments were performed: (i) lyophilized hydrogel rods with different F127DA concentrations (5%, 7.5%, 10%, 15%, and 20%, *w*/*v*); and (ii) 7.5% F127DA hydrogel rods stored at room temperature or lyophilized for 48 h. All samples were placed in PBS (pH 6.0) at 37 °C to simulate the static bladder environment. Every 4 h, the samples were vortexed for 10 s to simulate urine voiding. The floating state was recorded using a camera at predetermined time points and monitored continuously for 48 h.

### 4.4. Swelling Test

To verify the pH-responsive swelling behavior of CLMA, the swelling behavior of hydrogel rods without crosslinker was first evaluated at different F127DA concentrations (7.5%, 10%, and 15%, *w*/*v*). The samples were placed in the same PBS solution and removed at predetermined time points (1.5, 3, 12, 24, and 48 h). After the surface liquid was gently removed with filter paper, the three-dimensional dimensions (length L and diameter D) were measured using a vernier caliper with an accuracy of 0.01 mm. The swelling ratio (SR) was calculated according to the following Equation (1):

(1) SR (%) = [(Vt − V0)/V0] × 100%

where Vt and V0 represent the volume of the hydrogel rod after and before swelling, respectively, and V = pi × (D/2)^2^ × L.

Because CLMA possesses both pH-responsive properties and crosslinking-enhancing capability, two experimental groups were designed. First, hydrogel rods containing the same amount of CLMA were immersed in 50 mL PBS buffers with different pH values (pH 4.0, 6.0, and 8.0) at 37 °C. Second, lyophilized hydrogels containing different CLMA concentrations (0% and 2%) were immersed in 50 mL PBS buffer at the same pH value (pH 6.0) at 37 °C. At predetermined time points (1.5, 3, 12, 24, and 48 h), the samples were removed, and the swelling ratio was calculated using the same procedure.

### 4.5. Rheological Characterization

The rheological properties of the hydrogels were characterized using a rotational rheometer, including determination of the linear viscoelastic region, modulus analysis, thermoresponsiveness, shear-thinning behavior, and simulated bladder cyclic loading. Amplitude sweep tests were performed at a constant angular frequency with a shear strain range of 0.0001–1%. The storage modulus (G′) and loss modulus (G″) were recorded to determine the linear viscoelastic region. The same procedure was used to compare the modulus differences between photocrosslinked and physically crosslinked samples. Temperature sweep tests were performed at constant shear strain and frequency by increasing the temperature from 20 to 40 °C, and changes in G′ and the corresponding transition temperature were recorded. Shear-thinning tests were conducted at 37 °C by logarithmically increasing the shear rate from 0.1 to 1000 s^−1^, and the apparent viscosity was recorded.

Based on the above results, two shear strain levels within the linear viscoelastic region were selected to simulate urine storage (low strain) and voiding (high strain). The cycle was repeated three times, and changes in modulus were monitored to evaluate the adaptability of the material under dynamic bladder conditions [30, 31].

### 4.6. Biocompatibility Evaluation

Fresh citrated whole blood was obtained from healthy New Zealand white rabbits with ethical clearance (Sun Yat-sen University, Approval No. 2025000290, 17 March 2025).

Hemocompatibility: Fresh whole blood (1 mL) was centrifuged at 2000 rpm for 5 min to isolate red blood cells. After discarding the supernatant, the red blood cells were washed three times with PBS until the supernatant became clear and colorless. The cells were then resuspended in 5 mL PBS to prepare a 5% red blood cell suspension. In 1.5 mL EP tubes, 500 μL of hydrogel extract collected at different time points and 500 μL of red blood cell suspension were added. A negative control group (500 μL PBS + 500 μL red blood cell suspension) and a positive control group (500 μL 0.1% Triton X-100 + 500 μL red blood cell suspension) were also prepared, with three replicates in each group. All samples were incubated at 37 °C for 2 h and centrifuged at 2000 rpm for 5 min. The supernatant was collected, and absorbance at 540 nm (OD_540_) was measured using a microplate reader. The hemolysis rate was calculated according to the following Equation (2):

(2) Hemolysis rate (%) = [(OD_treatment_ − ODPBS)/(OD_Triton X-100_ − ODPBS)] × 100%

where OD_treatment_ is the OD_540_ value of the extract collected at different time points, and ODPBS and OD_Triton X-100_ are the OD_540_ values of the PBS-treated group and 0.1% Triton X-100-treated group, respectively.

Cell viability assay: Primary mouse bladder epithelial cells (mBECs) were seeded into 96-well plates at a density of 5000 cells per well. The 96-well plates and T25 culture flasks were precoated with rat tail collagen. Each well was supplemented with 200 μL complete medium for mouse bladder transitional epithelial cells, and the cells were cultured at 37 °C in a 5% CO_2_ incubator for 24 h to allow full attachment. After 24 h, the original medium was removed. The experimental groups received 150 μL fresh medium and 50 μL hydrogel extract collected at different time points, followed by incubation for another 24 h. The control group received 150 μL fresh medium and 50 μL PBS. For the CCK-8 assay, the old medium was removed, and 100 μL CCK-8 working solution (CCK-8 reagent diluted 1:10 with fresh medium) was added to each well. After incubation at 37 °C for 20 min, the supernatant was collected, and the absorbance at 450 nm (OD_450_) was measured using a microplate reader. The OD_450_ value of the CCK-8 working solution was used as the blank. Cell viability was calculated according to the following Equation (3):

(3) Cell viability (%) = [(OD_treatment_ − OD_blank_)/(OD_control_ − OD_blank_)] × 100%

where OD_treatment_, OD_control_, and OD_blank_ represent the OD_450_ values of the extract-treated group, control group, and blank group, respectively.

Live/dead staining: Primary mouse bladder epithelial cells (mBECs) were seeded into 96-well plates at a density of 5000 cells per well. The plates and T25 culture flasks were precoated with rat tail collagen. Each well was supplemented with 200 μL complete medium for mouse bladder transitional epithelial cells, and the cells were cultured at 37 °C in a 5% CO_2_ incubator for 24 h to allow full attachment. After 24 h, the original medium was removed. The experimental groups received 150 μL fresh medium and 50 μL hydrogel extract collected at different time points and were incubated for another 24 h. The control group received 150 μL fresh medium and 50 μL PBS. For staining, the old medium was removed, and each well was washed with PBS to remove residual medium. Then, 100 μL staining working solution containing PI and AM (prepared by adding 5 μL AM and 15 μL PI to 15 mL PBS) was added to each well, followed by incubation in a cell incubator at 37 °C for 20 min. After staining, the dye was discarded, and the cells were washed three times with PBS. Images were acquired using a multifunctional cell imaging microplate detection system.

### 4.7. Antibacterial Assay

*Escherichia coli* suspension (10 μL) was inoculated into 5 mL fresh TSB medium and cultured in a shaking incubator at 37 °C for 15 h. The bacterial concentration was determined by plate spreading and diluted with sterile TSB medium to 1 × 10^5^ CFU/mL. Cylindrical hydrogel samples prepared using molds were mixed with the diluted bacterial suspension and transferred into 48-well plates as the experimental groups. Drug-free floating hydrogels were used as the control group. All samples were incubated in a shaking incubator at 37 °C. Samples were collected at 24, 48, 72, and 96 h, and the absorbance at 600 nm (OD_600_) was measured using a microplate reader. The bacterial growth inhibition rate was calculated according to the following Equation (4):

(4) Inhibition rate (%) = [1 − (OD_treatment_ − OD_blank_)/(OD_control_ − OD_blank_)] × 100%

## Data Availability

Data available on request from the authors.

## Supplementary Materials

The following supporting information can be downloaded at: https://www.mdpi.com/article/10.3390/gels12080663/s1, Figure S1. Synthetic route and NMR characterization of F127DA. Figure S2. Quantitative data on hydrogel floatation. Figure S3. Synthetic route and ^1^H NMR characterization of CLMA. Figure S4. Drug loading and release properties of hydrogels. Figure S5. Mathematical fitting models of the drug release profiles from 7.5% F127DA/1% CLMA hydrogels at pH 6.0.

## Abbreviations

The following abbreviations are used in this manuscript:

CLMA	methacrylate-based crosslinker
IDD	intravesical drug delivery
iFDDS	intravesical floating drug delivery system
F127DA	Diacrylate-terminated Pluronic F127
PEO-PPO-PEO	poly(ethylene oxide)-poly(propylene oxide)-poly(ethylene oxide)

## References

[1] Mengistu D.A., Alemu A., Abdukadir A.A., Mohammed Husen A., Ahmed F., Mohammed B. (2023). Incidence of Urinary Tract Infection Among Patients: Systematic Review and Meta-Analysis. Inq. J. Health Care Organ. Provis. Financ..

[2] Flores-Mireles A.L., Walker J.N., Caparon M., Hultgren S.J. (2015). Urinary tract infections: Epidemiology, mechanisms of infection and treatment options. Nat. Rev. Microbiol..

[3] Ochoa D.C., Healy R. (2024). Intravesical Agents for Prevention of Recurrent Urinary Tract Infections. Eur. Urol. Focus.

[4] Foxman B. (2014). Urinary Tract Infection Syndromes Occurrence, Recurrence, Bacteriology, Risk Factors, and Disease Burden. Infect. Dis. Clin. N. Am..

[5] Morris C.J., Rohn J.L., Glickman S., Mansfield K.J. (2023). Effective Treatments of UTI—Is Intravesical Therapy the Future?. Pathogens.

[6] Patel D. (2024). Intravesical Therapies for Recurrent Urinary Tract Infections: A Systematic Review. Cureus.

[7] Kwon M., Ahmad A., Lott N., Blatt A. (2026). Intravesical therapy for recurrent urinary tract infection: A systematic review and meta-analysis. BJU Int..

[8] Gupta K., Hooton T.M., Naber K.G., Wullt B., Colgan R., Miller L.G., Moran G.J., Nicolle L.E., Raz R., Schaeffer A.J. (2011). International Clinical Practice Guidelines for the Treatment of Acute Uncomplicated Cystitis and Pyelonephritis in Women: A 2010 Update by the Infectious Diseases Society of America and the European Society for Microbiology and Infectious Diseases. Clin. Infect. Dis..

[9] Banerjee A., Lee D., Jiang C., Wang R., Kutulakos Z.B., Lee S., Gao J., Joshi N. (2024). Progress and challenges in intravesical drug delivery. Expert Opin. Drug Deliv..

[10] GuhaSarkar S., Banerjee R. (2010). Intravesical drug delivery: Challenges, current status, opportunities and novel strategies. J. Control Release.

[11] Khandelwal P., Abraham S., Apodaca G. (2009). Cell biology and physiology of the urothelium. Am. J. Physiol. Ren. Physiol..

[12] Qiu H., Guo H., Li D., Hou Y., Kuang T., Ding J. (2020). Intravesical Hydrogels as Drug Reservoirs. Trends Biotechnol..

[13] Li Z.A., Wen K.C., Liu J.H., Zhang C., Zhang F., Li F.Q. (2024). Strategies for intravesical drug delivery: From bladder physiological barriers and potential transport mechanisms. Acta Pharm. Sin. B.

[14] Wang S., Jin S., Shu Q., Wu S. (2021). Strategies to Get Drugs across Bladder Penetrating Barriers for Improving Bladder Cancer Therapy. Pharmaceutics.

[15] Zhang C., Niu J., Li J., Zhang H., Yu Q., Chen Y., Liu Y. (2024). Polysaccharide based supramolecular injectable hydrogels for in situ treatment of bladder cancer. Chin. Chem. Lett..

[16] Guo X., Li J., Wu Y., Xu L. (2024). Recent advancements in hydrogels as novel tissue engineering scaffolds for dental pulp regeneration. Int. J. Biol. Macromol..

[17] Wu Y., Gu X., Chen X., Cui Y., Jiang W., Liu B. (2024). Hydrogel: A new material for intravesical drug delivery after bladder cancer surgery. J. Mater. Chem. B.

[18] Arora S., Ali J., Ahuja A., Khar R.K., Baboota S. (2005). Floating drug delivery systems: A review. AAPS PharmSciTech.

[19] Singh B., Kim K. (2000). Floating Drug Delivery Systems: An Approach to Oral Controlled Drug Delivery via Gastric Retention. J. Control Release.

[20] Lin T., Wu J., Zhao X., Lian H., Yuan A., Tang X., Zhao S., Guo H., Hu Y. (2014). In Situ Floating Hydrogel for Intravesical Delivery of Adriamycin Without Blocking Urinary Tract. J. Pharm. Sci..

[21] Shriky B., Kelly A., Isreb M., Babenko M., Mahmoudi N., Rogers S., Shebanova O., Snow T., Gough T. (2020). Pluronic F127 thermosensitive injectable smart hydrogels for controlled drug delivery system development. J. Colloid Interface Sci..

[22] Lin T., Zhang Y., Wu J., Zhao X., Lian H., Wang W., Guo H., Hu Y. (2014). A Floating Hydrogel System Capable of Generating CO_2_ Bubbles to Diminish Urinary Obstruction After Intravesical Instillation. Pharm. Res..

[23] Zhu G., Zhang Y., Wang K., Zhao X., Lian H., Wang W., Wang H., Wu J., Hu Y., Guo H. (2015). Visualized intravesical floating hydrogel encapsulating vaporized perfluoropentane for controlled drug release. Drug Deliv..

[24] Yan Y., Song J., Liu D., Liu Z., Cheng J., Chen Z., Yang Y., Jiang W., Wang H., Ye J. (2024). Simple and Versatile in Situ Thermo-Sensitive Hydrogel for Rectal Administration of SZ-A to Alleviate Inflammation and Repair Mucosal Barrier in Ulcerative Colitis. Chin. Chem. Lett..

[25] Lin T., Zhao X., Zhang Y., Lian H., Zhuang J., Zhang Q., Chen W., Wang W., Liu G., Guo S. (2016). Floating Hydrogel with Self-Generating Micro-Bubbles for Intravesical Instillation. Materials.

[26] Kumari P., Goyal A.K. (2024). Challenges and opportunities in intravesical drug delivery approaches for the treatment of lower urinary diseases. J. Drug Deliv. Sci. Technol..

[27] Fu K., Wu H., Su Z. (2021). Self-assembling peptide-based hydrogels: Fabrication, properties, and applications. Biotechnol. Adv..

[28] Batrakova E., Kabanov A. (2008). Pluronic block copolymers: Evolution of drug delivery concept from inert nanocarriers to biological response modifiers. J. Control Release.

[29] Highley C.B., Rodell C.B., Burdick J.A. (2015). Direct 3D Printing of Shear-Thinning Hydrogels into Self-Healing Hydrogels. Adv. Mater..

[30] Fu Z., Xiao S., Wang P., Zhao J., Ling Z., An Z., Shao J., Fu W. (2023). Injectable, stretchable, toughened, bioadhesive composite hydrogel for bladder injury repair. RSC Adv..

[31] Loebel C., Rodell C.B., Chen M.H., Burdick J.A. (2017). Shear-thinning and self-healing hydrogels as injectable therapeutics and for 3D-printing. Nat. Protoc..

