# Supplementary material for: Catheter-Deliverable Floating Hydrogels for Sustained Intravesical Drug Release
Source: Gels. 2026 Jul 23;12(8):663. doi: 10.3390/gels12080663 (PMC13512294; doi:10.3390/gels12080663)
Supplement: Supplementary file 1 [file gels-12-00663-s001.zip › gels-4432775-supplementary.pdf]

Catheter-deliverable Floating Hydrogels for Sustained Intravesical Drug Release

Jing Li[1†], Chao Ni[1†], Sitian Li[1], Yutian Huang[1], Jun Yue[1,2*]

[1]School of Biomedical Engineering, Shenzhen Campus of Sun Yat-sen University, Shenzhen, Guangdong 518107, P. R. China.

[2] NMPA Center for Innovation and Research in Regulatory Science, School of Biomedical Engineering, Shenzhen Campus of Sun Yat-sen University, Shenzhen, Guangdong, 518107, P. R. China.

[*]Corresponding to: yuejun3@mail.sysu.edu.cn

[†]These authors contributed equally.

Content

Synthesis of F127DA 2

Synthesis of CLMA 2

^{1}H NMR characterizations 3

Determination of drug loading and encapsulation efficiency 3

Drug release based on dialysis 4

Drug release investigation using a bladder simulation model 5

Figure S1. Synthetic route and NMR characterization of F127DA. 6

Figure S2. Quantitative data on hydrogel floatation. 7

Figure S3. Synthetic route and ^{1}H NMR characterization of CLMA. 8

Figure S4. Drug loading and release properties of hydrogels. 8

Figure S5. Mathematical fitting models of the drug release profiles from 7.5% F127DA/1% CLMA hydrogels at pH 6.0. 9

Synthesis of F127DA

F127DA was synthesized according to a previously reported method. Briefly, 10.00 g of Pluronic F127 (Mn ≈ 12,600 g/mol) was dissolved in 100 mL of anhydrous dichloromethane in a three-neck round-bottom flask. The solution was cooled in an ice bath under a nitrogen atmosphere. Subsequently, triethylamine and acryloyl chloride, each at a 10-fold molar excess relative to the terminal hydroxyl groups of F127, were added dropwise while maintaining the reaction temperature below 30 °C. After complete addition, the ice bath was removed, and the reaction mixture was stirred continuously at room temperature for 24 h.

Upon completion, the precipitated triethylamine hydrochloride was removed by filtration. The filtrate was then slowly added dropwise into an excess amount of pre-cooled diethyl ether (−20 °C) to precipitate the product. This purification procedure was repeated three times to remove residual monomers and by-products thoroughly. The resulting white solid was vacuum-dried at 25 °C for 24 h to obtain highly purified F127DA, which was stored at room temperature protected from light until further use.

Synthesis of CLMA

The small-molecule crosslinker CLMA was synthesized following a previously reported procedure. Briefly, 1,4-N,N′-bis(3-aminopropyl)piperazine (2 g, 10 mmol) was dissolved in 20 mL of anhydrous dichloromethane in a dry 50 mL round-bottom flask and cooled to 0 °C in an ice bath. Under a nitrogen atmosphere, methacrylic anhydride (3.23 g, 21 mmol) dissolved in 5 mL of anhydrous dichloromethane was added dropwise to the reaction system. A catalytic amount of triethylamine (approximately 0.5 mL) was added

simultaneously. After the addition was complete, the ice bath was removed and the reaction mixture was stirred at room temperature for 24 h.

The reaction mixture was adjusted to pH 9–10 using 0.1 M NaOH solution and washed three times with saturated brine (3 × 30 mL). The lower organic phase was collected and dried over anhydrous magnesium sulfate for 3 h, followed by filtration. The solvent was removed by rotary evaporation. The obtained pale-yellow solid was vacuum-dried at 50 °C for 15 h to afford the dimethacrylamide product.

^{1}H NMR characterizations

For ^{1}H NMR analysis, 5 mg of synthesized F127DA and MA-BAP-MA were separately dissolved in 600 μL of deuterated chloroform ($CDCl_3$). The solutions were transferred into NMR tubes and analyzed using a 400 MHz NMR spectrometer. The chemical structures of the synthesized products were confirmed by evaluating the chemical shifts and integral areas of characteristic proton signals.

Determination of drug loading and encapsulation efficiency

Rifampicin (RFP) standard solutions with concentrations of 1, 5, 10, 25, 50, and 100 μg/mL were prepared using phosphate-buffered saline (PBS) at pH 4.0, 6.0, and 8.0. The absorbance of each solution was measured at 337 nm using a UV–Vis spectrophotometer. Standard calibration curves were established by plotting drug concentration (C, μg/mL) against absorbance (Abs) and performing least-squares linear regression analysis. All calibration curves exhibited excellent linearity ($R^2 \geq 0.999$):

$$\text{pH 4.0: Abs} = 0.021C + 0.0159 \ (R^2 = 0.999, n = 3)$$

$$\text{pH 6.0: Abs} = 0.0383C + 0.0029\ (R^2 = 1.000,\ n = 3)$$

$$\text{pH 8.0: Abs} = 0.0351C + 0.0047\ (R^2 = 0.999,\ n = 3)$$

A predetermined amount of drug-loaded hydrogel was immersed in 5 mL of PBS (pH 6.0) and sonicated for 1 h to accelerate drug release from the hydrogel matrix. The solution was subsequently transferred into a dialysis bag (MWCO 1000 Da) and dialyzed against 20 mL of PBS (pH 6.0) for 24 h.

The absorbance of the dialysate was measured at the characteristic UV absorption wavelength of rifampicin. The rifampicin content in the hydrogel was calculated using the corresponding calibration curve. Drug loading (DL) and encapsulation efficiency (EE) were subsequently determined according to the following equations:

$$\text{DL (\%)} = (\text{Mass of drug in gel} / \text{Total mass of drug-loaded gel}) \times 100\%$$

$$\text{EE (\%)} = (\text{Mass of drug in gel} / \text{Total mass of drug fed}) \times 100\%$$

Drug release based on dialysis

Drug-loaded hydrogel rods were accurately weighed and placed into pretreated dialysis bags (MWCO 1000 Da; boiled for 10 min before use). The dialysis bags were immersed separately in 20 mL of PBS release media with different pH values (4.0, 6.0, and 8.0). The release systems were maintained in a thermostatic shaking incubator at 37 °C and 100 rpm.

At predetermined time intervals (0, 0.5, 1, 2, 6, 12, 24, and 48 h), 3 mL of release medium was withdrawn and replaced with an equal volume of fresh medium. After filtration through a 0.22 μm membrane, the absorbance at 337 nm was measured. The cumulative release percentage (Q) was calculated using the following equation:

$$Q_t (\%) = [C_n \times V + \Sigma(i=1 \text{ to } n-1)(C_i \times V_i)] / W_0 \times 100\%$$

where C_n is the drug concentration at time t , V_i is the volume of release medium, C_i and V_i are the concentration and sampling volume at previous time points, respectively, and W_0 is the initial drug mass in the gel.

Drug release investigation using a bladder simulation model

Considering the physiological voiding behavior of the urinary bladder, an in vitro bladder simulation model was further established to evaluate drug release performance. Drug-loaded hydrogels were placed in a 100 mL beaker. Artificial urine medium was continuously introduced into the beaker using a peristaltic pump at a flow rate of 20 mL/h. Every 4 h, the liquid in the beaker was slowly removed until approximately 5 mL remained. This filling-and-emptying process was repeated for three cycles to simulate the physiological bladder filling and voiding process. A single administration of liquid drug formulation was used as the control group. Samples (3 mL) were collected every hour and replaced with an equal volume of fresh artificial urine medium. The cumulative drug release during each cycle was calculated using the same equation described above.

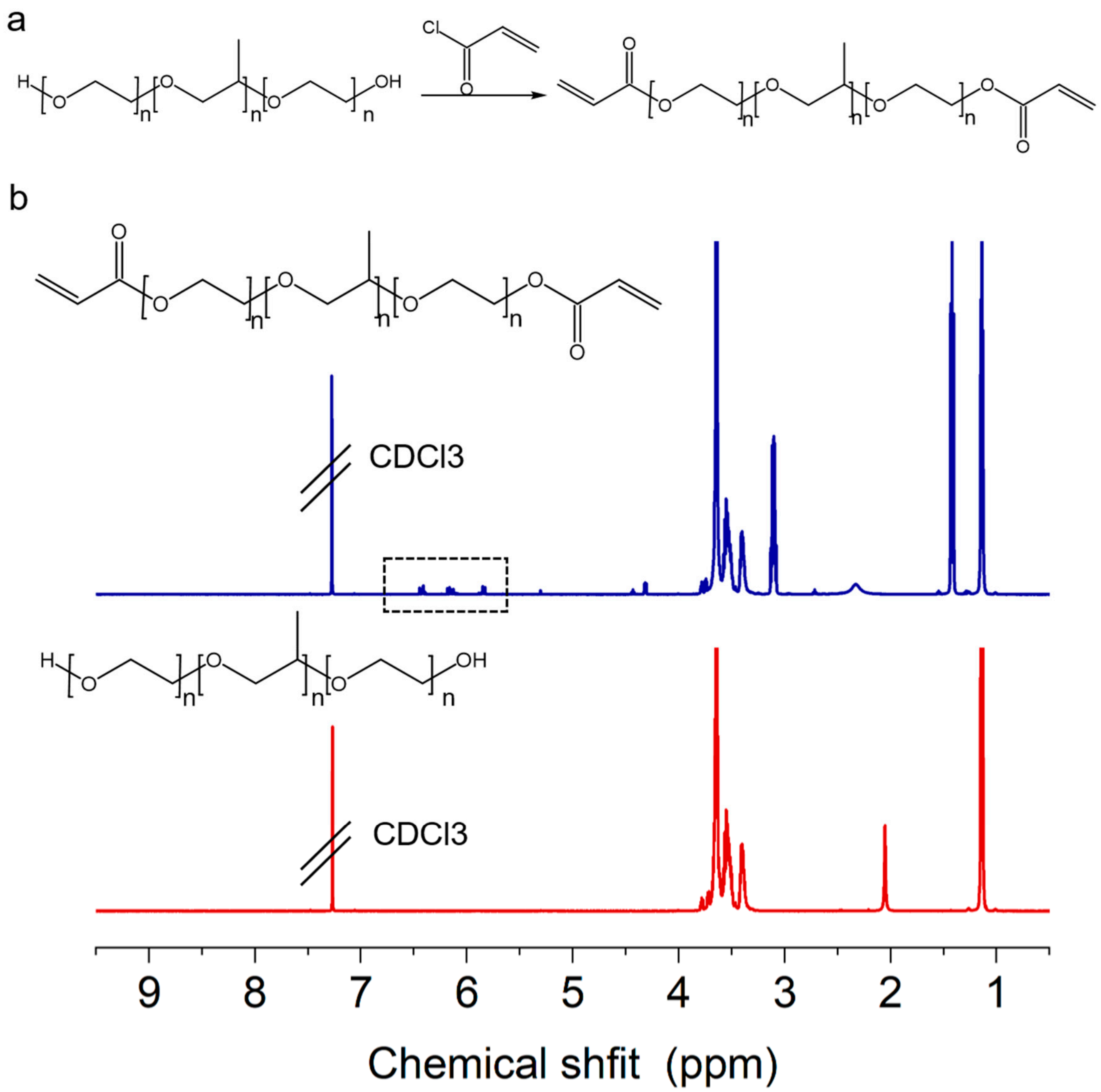

Figure S1. Synthetic route and NMR characterization of F127DA.

(a) Schematic illustration of the synthetic route of F127DA. F127 was functionalized with methacrylate groups to introduce double bonds at both terminals. (b) Comparative 1H NMR spectra of F127DA and pristine F127. The appearance of three vinyl proton peaks in the range of 5.5–6.5 ppm confirms the successful synthesis of F127DA.

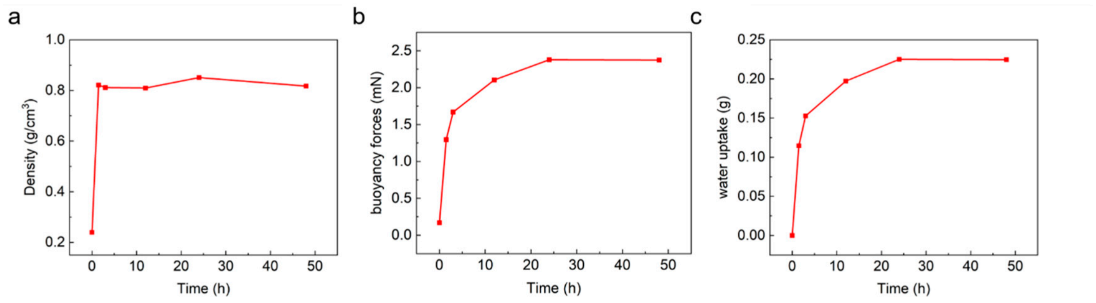

Figure S2. Quantitative data on hydrogel floatation.

(a) Apparent density changes; (b) real-time buoyant force; (c) water absorption rate curves of the hydrogel rods.

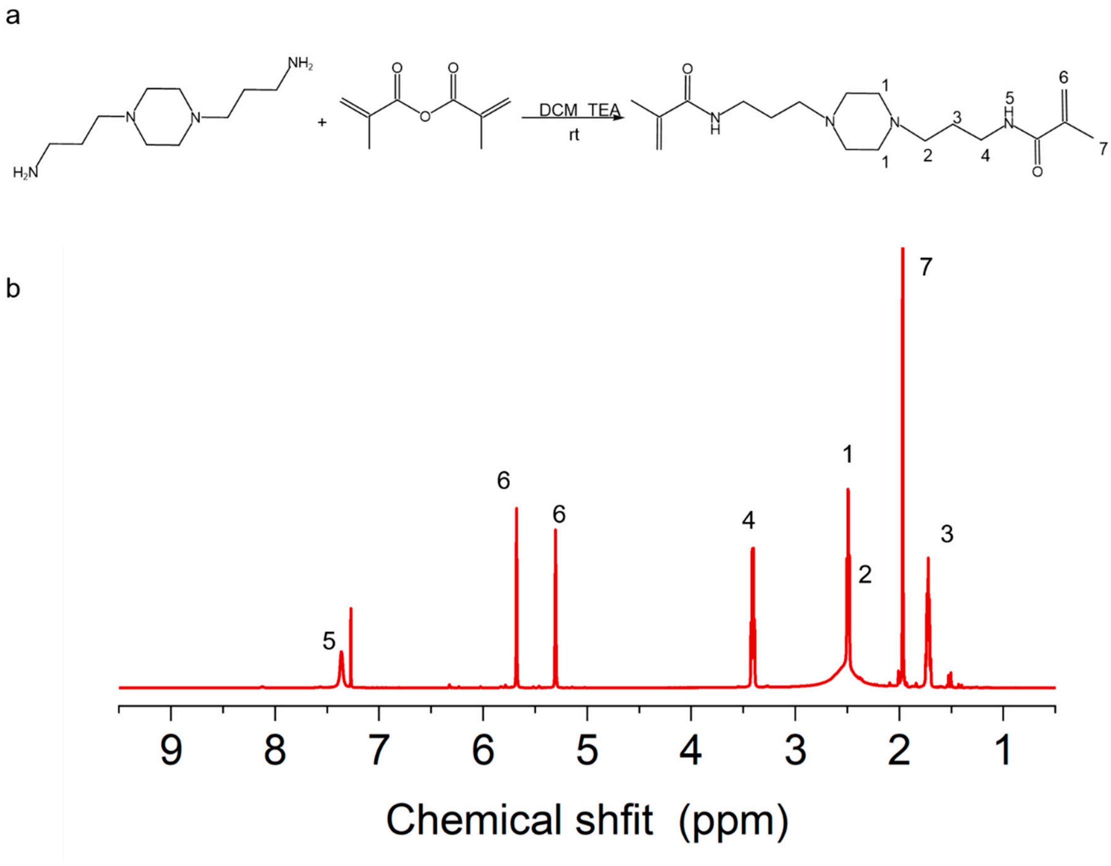

Figure S3. Synthetic route and ¹H NMR characterization of CLMA.

(a) Schematic illustration of the synthetic route of CLMA. The crosslinker was synthesized via the reaction of an amine with methacrylic anhydride, yielding a molecule featuring two tertiary amine groups and a terminal double bond. (b) 1H NMR spectrum of the synthesized CLMA. The peak assignments correspond precisely to the chemical structure, confirming the successful synthesis of the small-molecule crosslinker.

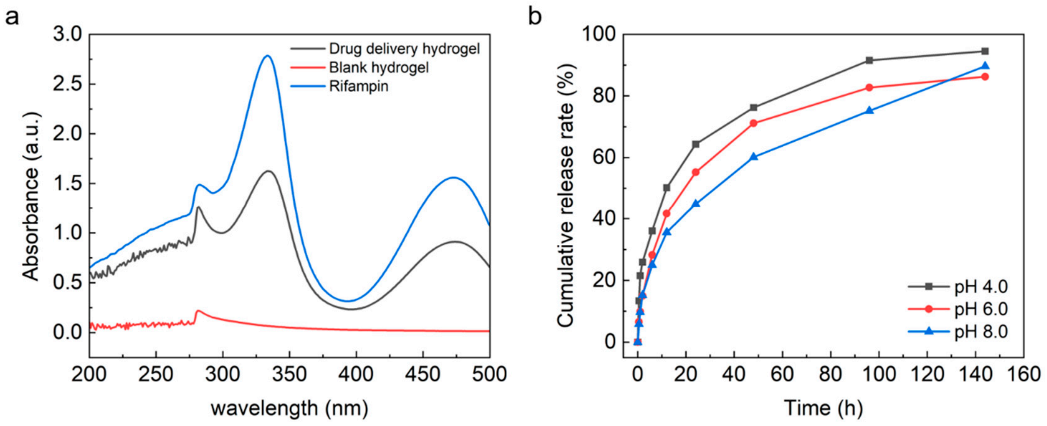

Figure S4. Drug loading and release properties of hydrogels.

(a) Rifampicin loading capacity of floating drug delivery systems; (b) pH-dependent cumulative drug release profiles from hydrogels comprising 7.5% F127DA and 1% CLMA.

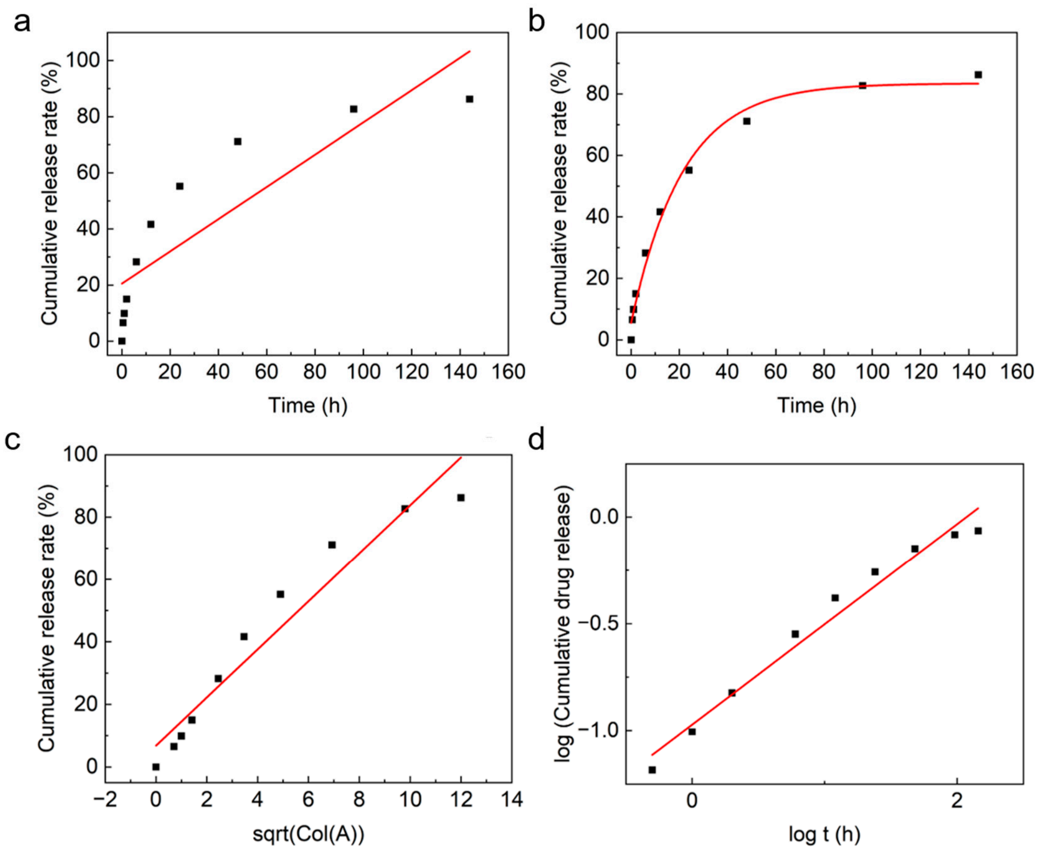

Figure S5. Mathematical fitting models of the drug release profiles from 7.5% F127DA/1% CLMA hydrogels at pH 6.0.

(a) Zero-order model; (b) first-order model; (c) Higuchi model; (d) Korsmeyer–Peppas model.
